# Prediction of Fluid Responsiveness by the Effect of the Lung Recruitment Maneuver on the Perfusion Index in Mechanically Ventilated Patients During Surgery

**DOI:** 10.3389/fmed.2022.881267

**Published:** 2022-06-17

**Authors:** Sunthiti Morakul, Naruemol Prachanpanich, Pattarada Permsakmesub, Pimwatana Pinsem, Wasineenart Mongkolpun, Konlawij Trongtrakul

**Affiliations:** ^1^Department of Anesthesiology, Faculty of Medicine Ramathibodi Hospital, Mahidol University, Bangkok, Thailand; ^2^Department of Critical Care Medicine, Siriraj Piyamaharajkarun Hospital, Siriraj Hospital, Mahidol University, Bangkok, Thailand; ^3^Department of Internal Medicine, Faculty of Medicine, Chiang Mai University, Chiang Mai, Thailand

**Keywords:** perfusion index (PI), fluid responsiveness, lung recruitment maneuver, mechanical ventilation, perioperative period

## Abstract

**Introduction:**

Excessive or inadequate fluid administration during perioperative period affects outcomes. Adjustment of volume expansion (VE) by performing fluid responsiveness (FR) test plays an important role in optimizing fluid infusion. Since changes in stroke volume (SV) during lung recruitment maneuver (LRM) can predict FR, and peripheral perfusion index (PI) is related to SV; therefore, we hypothesized that the changes in PI during LRM (ΔPI_LRM_) could predict FR during perioperative period.

**Methods:**

Patients who were scheduled for elective non-laparoscopic surgery under general anesthesia with a mechanical ventilator and who required VE (250 mL of crystalloid solution infusion over 10 min) were included. Before VE, LRM was performed by a continuous positive airway pressure of 30 cm H_2_O for 30 sec; hemodynamic variables with their changes (PI, obtained by pulse oximetry; and ΔPI_LRM_, calculated by using [(PI before LRM—PI after LRM)/PI before LRM]^*^100) were obtained before and after LRM. After SV (measured by esophageal doppler) and PI had returned to the baseline values, VE was infused, and the values of these variables were recorded again, before and after VE. Fluid responders (Fluid-Res) were defined by an increase in SV ≥10% after VE. Receiver operating characteristic curves of the baseline values and ΔPI_LRM_ were constructed and reported as areas under the curve (AUC) with 95% confidence intervals, to predict FR.

**Results:**

Of 32 mechanically ventilated adult patients included, 13 (41%) were in the Fluid-Res group. Before VE and LRM, there were no differences in the mean arterial pressure (MAP), heart rate, SV, and PI between patients in the Fluid-Res and fluid non-responders (Fluid-NonRes) groups. After LRM, SV, MAP, and, PI decreased in both groups, ΔPI_LRM_ was greater in the Fluid-Res group than in Fluid-NonRes group (55.2 ± 17.8% vs. 35.3 ± 17.3%, *p* < 0.001, respectively). After VE, only SV and cardiac index increased in the Fluid-Res group. ΔPI_LRM_ had the highest AUC [0.81 (0.66–0.97)] to predict FR with a cut-off value of 40% (sensitivity 92.3%, specificity 73.7%).

**Conclusions:**

ΔPI_LRM_ can be applied to predict FR in mechanical ventilated patients during the perioperative period.

## Introduction

Perioperative fluid administration has a crucial role during perioperative management. Both excessive (1–3) and insufficient fluid infusion (4, 5) are related to poor outcomes including the development of organ dysfunction or death in patients undergoing abdominal surgery. The benefit of hemodynamic parameters such as cardiac output (CO) or stroke volume (SV)-guided fluid infusion on mortality or postoperative complication such as surgical site infection, acute kidney injury, has been demonstrated in recent meta-analyses (6, 7). However, this benefit seems to be limited in high-risk surgical patients (8, 9). Nevertheless, in the FEDORA trial (10), the advantage of CO-guided volume expansion (VE) or vasopressor titration on the development of acute kidney injury or pulmonary edema during post operative period in low-to-moderate-risk surgical patients undergoing major abdominal surgery has been demonstrated.

Dynamic parameters such as pulse pressure variation (PPV) or stroke volume variation (SVV) predict fluid responsiveness better than static parameters such as mean arterial pressure (MAP) or central venous pressure (CVP) (11–13). Nevertheless, the abilities of PPV or SVV to predict fluid responsiveness in patients with either open abdominal wall (14), abdominal hypertension (15), or in surgical patients during general anesthesia (16) are limited. Cannesson et al. (16) demonstrated inconclusive evidence of the ability of PPV to detect fluid responsiveness in ~25% of patients during general anesthesia. However, to measure the dynamic change of PPV and SVV during a transient increase in the intrathoracic pressure lung recruitment maneuver (LRM) (17, 18) or tidal volume challenge (19–21) improved the accuracy of PPV or SVV to predict fluid responsiveness. Nevertheless, PPV or SVV require arterial catheter insertion with its inherent risk (22); therefore, a non-invasive measure such as pleth variation index (PVI) using pulse oximetry might be an alternative measurement (23–25).

Peripheral perfusion index (PI), which shows the ratio between pulsatile and non-pulsatile portions, is obtained using pulse oximetry, similar to PVI, which is a measure of the dynamic changes in PI that occur during one or more complete respiratory cycles. PI depends on SV, CO, and peripheral vascular tone (26, 27). Therefore, PI can be used to track changes in the systemic hemodynamic parameters (28). However, studies reporting changes in PI during LRM in surgical patients are limited. Therefore, in this study, we hypothesized that PI would be reduced during LRM and this change might predict fluid responsiveness in surgical patients.

## Materials and Methods

### Study Design

This prospective diagnostic study was conducted in operating rooms at the Faculty of Medicine, Ramathibodi Hospital, Bangkok, Thailand, from November 2020 to April 2021. The study protocol was approved by the local ethical committee (approval number COA. MURA2020/1844). The informed consent was obtained from each patient on the day before surgery. Patients were included if they were aged ≥18 years, were scheduled for elective non-laparoscopic surgery under general anesthesia with a controlled mechanical ventilation, and required their first VE during perioperative period. We excluded patients who had uncontrolled hemodynamic status (29, 30), intracranial hypertension, severe chronic obstructive pulmonary disease, broncho alveolar fistula, severe emphysema, and those with pre-existing comorbidities including severe left and right ventricular dysfunction, severe pulmonary hypertension (30, 31), severe obesity (BMI >40 kg/m^2^), and pregnancy.

After anesthesia induction, an endotracheal tube and arterial catheter were placed in all included patients. The dose or type of anesthesia agents and an anesthesia mechanical ventilator were managed by the attending anesthesiologists. An anesthesia machine ventilator was set to achieve a low tidal volume (6–8 mL/kg predicted body weight, aiming for an expired ratio of 1:2), positive end-expiratory pressure (PEEP) of 3–5 cm H_2_O, respiratory rate that was adjusted to obtain an appropriate end-tidal carbon dioxide (EtCO_2_) amount between 30 and 35 mmHg, and an inspiratory oxygen fraction (FiO_2_) was set to achieve an SpO_2_ of at least 95%.

**Figure 1:**
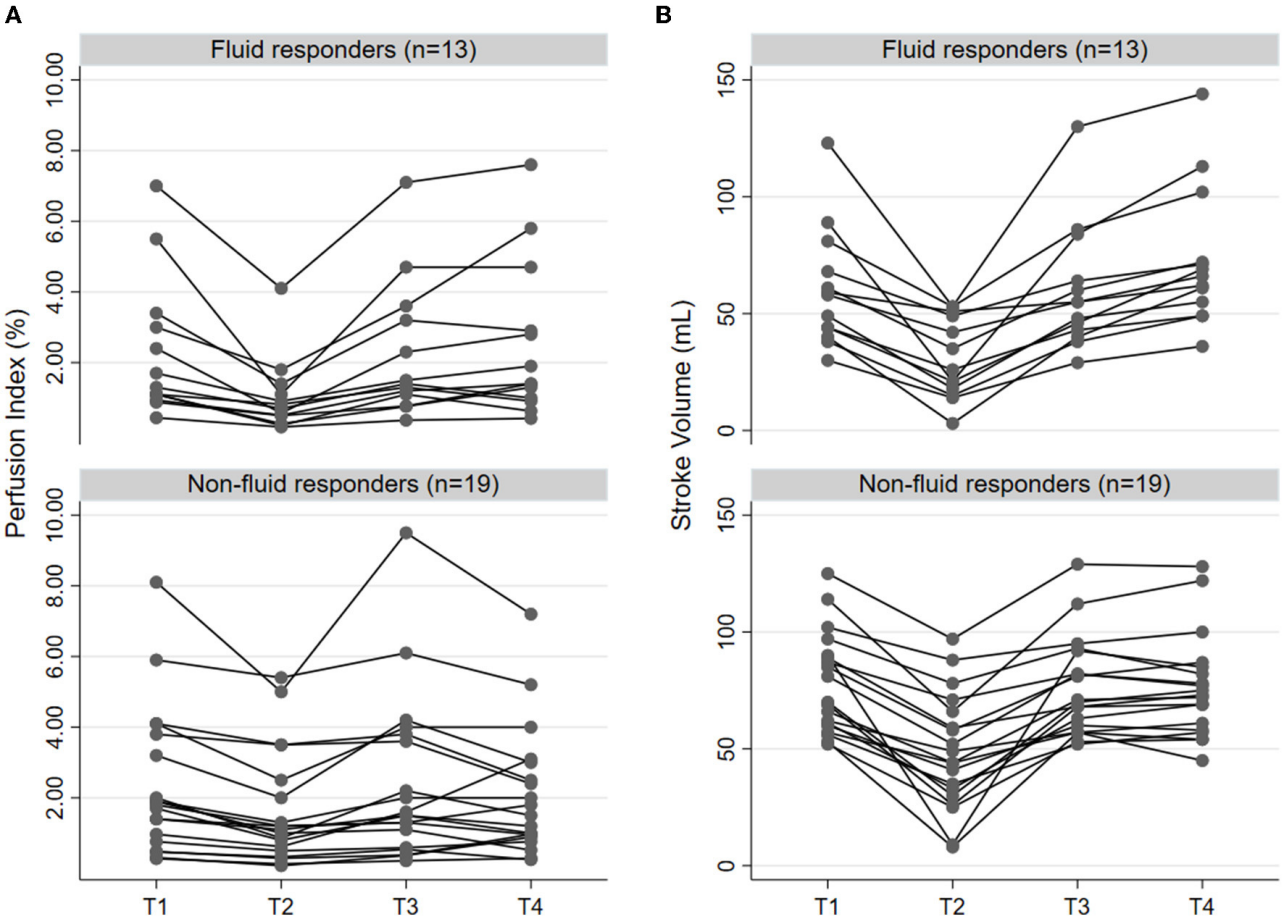
Individual change of prefusion index **(A)** and stroke volume **(B)** according to the status of fluid responsiveness (*n* = 13) and non-responsiveness (*n* = 19) in four time points, including before LRM (T1), after LRM (T2), before VE (T3), and immediately after VE (T4). LRM, lung recruitment maneuver; VE, volume expansion.

Demographic data were recorded from medical record. Continuous blood pressure, continuous electrocardiogram, heart rate (HR), EtCO_2_, and SpO_2_ (measured by pulse oximetry), were monitored during the perioperative period. VE, defined as 250 mL of crystalloid solution infusion over 10 min, was decided by the attending physicians. Esophageal Doppler probe (DCQ ODM, Deltex, Chichester, Sussex, UK) and PI (on the third or fourth finger) were placed before the initiation of LRM until the end of VE. The esophageal Doppler probe was positioned to attain the best aortic blood velocity signal. LRM had been performed before surgery began and thus with closed abdomen by applying a continuous positive airway pressure (CPAP) of 30 cm H_2_O for 30 s before VE was infused. Systolic blood pressure (SBP), diastolic blood pressure (DBP), MAP, CO, SV, PVV, SVV, PVI, and PI were obtained before LRM as the first baseline values (T1) and after LRM (T2). After SV and PI returned to their baseline values (variations <10%), hemodynamic variables and PI were obtained as the second baseline values (T3); then, VE was infused and these hemodynamic variables and PI were recorded immediately after VE (T4) [Supplemental Digital Content (SDC), Figure 1]. Changes in hemodynamic variables and PI during LRM and VE were recorded and presented as relative percent change from the baseline value before LRM and VE, respectively. These were calculated using the following formula:

Before (T1) vs. after LRM (T2)

Relative change of SV (ΔSV_LRM_) = ([SV_T1_- SV_T2_]/SV_T1_)^*^100
Relative change of CO (ΔCO_LRM_) = ([CO_T1_- CO_T2_]/CO_T1_)^*^100
Relative change of MAP (ΔMAP_LRM_) = ([MAP_T1_- MAP_T2_]/MAP_T1_)^*^100
Relative change of PPV (ΔPPV_LRM_) = ([PPV_T2_- PPV_T1_]/PPV_T1_)^*^100
Relative change of PVI (ΔPVI_LRM_) = ([PVI_T2_- PVI_T1_]/PVI_T1_)^*^100

Before (T3) vs. after VE (T4)

Relative change of SV (ΔSV_VE_) = ([SV_T4_-SV_T3_]/SV_T3_)^*^100
Relative change of CO (ΔCO_VE_) = ([CO_T4_- CO_T3_]/CO_T3_)^*^100
Relative change of MAP (ΔMAP_VE_) = ([MAP_T4_- MAP_T3_]/MAP_T3_)^*^100

SV, SVV, and CO were derived from the esophageal Doppler. PPV was derived from the Philips^®^ IntelliVue MP 50 monitor. All the patients were grouped according to whether they were fluid responders or not, which was defined by an increase in SV (obtained by esophageal Doppler) ≥10% after VE. All the patients were included once.

### PI Measurements

PI, a unit expressed as a percentage, was measured using a pulse oximeter, the Radical-7 Pulse CO-Oximeter device (Masimo Corporation, Irvine, CA, USA) with an adult disposable spectrophotometric sensor, ReSposable^TM^ R2–25 (Masimo Corporation, Irvine, CA, USA). The PI was calculated as the ratio of the pulsatile over non-pulsatile amplitudes detected by the sensor. The short-time method was used to display the PI values during LRM. A percent decrease in PI according to the LRM (ΔPI_LRM_) was calculated using this formula:

Relative change of PI (ΔPI_LRM_) = ([PI_T1_- PI_T2_]/PI_T1_)^*^100

### Sample Size Calculation

The sample size was calculated based on the assumption that ΔPI_LRM_ could determine fluid responsiveness at an AUC of 0.80, corresponding to a good discriminative ability for the diagnostic test. The null hypothesis value of AUC was set at 0.50. The proportion of fluid responders was 45%, corresponding to a fluid responsiveness ratio (negative-to-positive) of 1.222. The risk of alpha error at 5% and beta error at 10% were accounted for. In total, 27 patients were needed. The sample size was calculated using Obuchowski's method (32) via a web tool for ROC curve analysis (version 1.3.1) (33). To counteract the 15% dropout rate, 32 patients were planned for inclusion.

### Statistical Analysis

Continuous data are expressed as the mean ± standard deviation (SD). Categorical data are expressed as counts (n) and percentages (%). The comparison of hemodynamic parameters before (T1) and after LRMs (T2) and before (T3) and after VE (T4) were performed using the paired *t*-test or Wilcoxon signed rank test as appropriate. Comparisons between fluid responders and non-responders were performed using the two-tailed Student *t*-tests or the Wilcoxon test as appropriate. The diagnostic performances of ΔPI_LRM_, ΔSV_LRM_, ΔCO_LRM_, and ΔMAP_LRM_ for detecting fluid responsiveness were estimated by the area under the receiver operating characteristic curves (AUCs). Sensitivity (Sn), specificity (Sp), positive predictive value, negative predictive value, positive likelihood ratio (LR+), and negative likelihood ratio (LR-) were reported accordingly. The best cut-off value was determined by the Youden Index (Sn + Sp−1).

The gray zone approach to identifying inconclusive ranges of ΔPI_LRM_ that could not determine whether the patients were fluid responders or non-responders, was constructed using two approaches. First, the bootstrap resampling of 1,000 replications was performed to identify the best cut-off point and its 95% confidence interval (CI) (representative of the gray zone). Second, the three class responses for ΔPI_LRM_, including inclusion, inconclusion, and exclusion, were determined. Thresholds related to the Sn <90% and Sp <90% were set. Then, the remaining area was deemed inconclusive or designated a gray zone. The larger size from the two approaches was used to indicate the gray zone (34, 35). Correlation was performed by linear correlation, according to the data distribution. A *p*-value < 0.05 was considered statistically significant. All analyses were performed using the STATA statistical software version 16.0 (StataCorp LP, College Station, Tx, USA).

## Results

### Patient Characteristics

The baseline characteristics of the 32 patients included in this study are shown in Table 1. Thirteen (40.6%) patients were fluid responders and 19 were not. There were no differences in baseline characteristics between fluid responders and fluid non-responders (SDC, Supplementary Table 1).

**Table 1:** Patient baseline characteristics (*n* = 32).

**Variables**	**Results**
Age (years)	60 ± 10
Male, *n* (%)	20 (62.5%)
Height (cm)	160.0 ± 6.8
Body weight (kg)	60.4 ± 12.1
Predicted body weight (kg)	55.3 ± 7.6
Body mass index (kg/m^2^)	23.5 ± 4.2
Body surface area (m^2^)	1.62 ± 0.16
**ASA physical status**, ***n*** **(%)**
I	2 (6.2%)
II	15 (46.9%)
III	15 (46.9%)
**Operative sites**, ***n*** **(%)**
Liver	14 (43.8%)
Pancreas	11 (34.4%)
Renal	3 (9.4%)
Gynecology	3 (9.4%)
Breast	1 (3.0%)
Tidal volume (mL)	480 ± 48
Tidal volume/predicted body weight (ml/kg)	8.7 ± 0.7
Respiratory rate (breaths/min)	12.8 ± 1.4
Plateau pressure (cm H_2_O)	16.1 ± 2.2
Driving pressure (cm H_2_O)	11.2 ± 2.2
Positive end expiratory pressure (cm H_2_O)	5.0 ± 1.0
Vasopressor usage, *n* (%)	5 (15.6%)
Sevoflurane, *n* (%)	22 (68.8%)
Desflurane, *n* (%)	10 (32.3%)

*ASA, American Society of Anesthesiologists*.

### The Effect of LRM on Hemodynamic Variables and PI in Fluid Responders vs. Non-responders

Before LRM, there were no differences in MAP, CO, PVI, PI between fluid responders and non-responders (Table 2). PVV and SVV were greater in fluid responders than in non-responders (Table 2). After LRM, CO, SV, MAP, PI reduced, while PVI, PPV, and SVV increased in both groups (Table 2). ΔPI_LRM_ were greater in fluid responders than in non-responders (55.2 ± 17.8% vs. 35.3 ± 17.3%, *p* = 0.004, respectively). ΔSV_LRM_ (49.4 ± 21.5% vs. 39.8 ± 21.4%, *p* = 0.222, respectively), ΔMAP_LRM_ (26.3 ± 10.9% vs. 19.5 ± 9.6%, *p* = 0.073, respectively), ΔPVI_LRM_ (45.3 ± 10.0% vs. 35.9 ± 6.4%, *p* = 0.410, respectively), and ΔPPV_LRM_ (58.1 ± 84.8% vs. 95.9 ± 102.5%, *p* = 0.297, respectively) did not differ between fluid responders and non-responders. Individual changes in PI and SV in fluid responders and non-responders are presented in Figure 1.

**Table 2:** Hemodynamic parameters before and after lung recruitment maneuver and volume expansion.

**Hemodynamic parameters**	**Lung recruitment maneuve**		***P*** **-value**	**Volume expansion**		***P*** **-value**
	**Before (T1)**	**After (T2)**		**Before (T3)**	**After (T4)**	
**Perfusion index**						
Fluid responders (*n* = 13)	2.30 ± 1.98	1.01 ± 1.04	0.002	2.25 ± 1.93	2.52 ± 2.21	0.197
Fluid non- responders (*n* = 19)	2.35 ± 2.08	1.65 ± 1.60	<0.001	2.41 ± 2.35	2.08 ± 1.81	0.110
**Stroke volume (mL)**						
Fluid responders (*n* = 13)	60.3 ± 25.4	30.8 ± 17.2	<0.001	59.8 ± 26.9	73.0 ± 29.8	<0.001
Fluid non-responders (*n* = 19)	77.7 ± 21.4^$^	48.1 ± 24.6^$^	<0.001	75.9 ± 21.0	76.1 ± 21.9	0.881
**Cardiac output (L/min)**						
Fluid responders (*n* = 13)	4.28 ± 1.30	1.78 ± 0.99	<0.001	4.25 ± 1.49	5.05 ± 1.84	<0.001
Fluid non-responders (*n* = 19)	5.13 ± 1.69	2.78 ± 1.61	<0.001	4.83 ± 1.56	4.95 ± 1.62	0.328
**Systolic blood pressure (mmHg)**						
Fluid responders (*n* = 13)	113 ± 18	78 ± 15	<0.001	108 ± 19	116 ± 16	0.013
Fluid non-responders (*n* = 19)	114 ± 18	92 ± 14	<0.001	107 ± 17	122 ± 24	<0.001
**Diastolic blood pressure (mmHg)**						
Fluid responders (*n* = 13)	64 ± 17	52 ± 13	<0.001	63 ± 15	63 ± 14	0.958
Fluid non-responders (*n* = 19)	61 ± 8	52 ± 10	<0.001	58 ± 8	66 ± 13	<0.001
**Mean arterial pressure (mmHg)**						
Fluid responders (*n* = 13)	82 ± 16	60 ± 13	<0.001	80 ± 15	83 ± 13	0.068
Fluid non-responders (*n* = 19)	81 ± 13	66 ± 15	<0.001	77 ± 11	88 ± 17	<0.001
**Heart rate (beats/min)**						
Fluid responders (*n* = 13)	75 ± 17	71 ± 15	0.051	75 ± 16	73 ± 15	0.325
Fluid non-responders (*n* = 19)	67 ± 14	62 ± 15	<0.001	64 ± 13^$^	65 ± 15	0.215
**Pleth variability index (%)**						
Fluid responders (*n* = 13)	13.9 ± 4.8	18.9 ± 4.2	<0.001	–	9.7 ± 3.2	–
Fluid non-responders (*n* = 19)	12.0 ± 6.8	15.5 ± 7.5	<0.001	–	12.6 ± 6.5	–
**Pulse pressure variation (%)**						
Fluid responders (*n* = 13)	18.8 ± 7.5	24.5 ± 7.4	<0.001	–	12.0 ± 8.2	–
Fluid non-responders (*n* = 19)	10.0 ± 5.8^**^	17.2 ± 6.9^*^	<0.001	–	6.0 ± 3.5^$^	–
**Stroke volume variation (%)**						
Fluid responders (*N* = 13)	25.2 ± 13.0	43.3 ± 14.0	0.003	–	18.4 ± 8.0	–
Fluid non-responders (*N* = 17)	17.9 ± 6.3^$^	27.3 ± 15.4^$^	0.036	–	18.8 ± 12.2	–

*Data are expressed as mean ± SD*.

$ *p < 0.05 comparing between fluid responders and non-responders at the same period of time*.

* *p < 0.01 comparing between fluid responders and non-responders at the same period of time*.

** *p < 0.001 comparing between fluid responders and non-responders at the same period of time*.

### The Effect of VE on Hemodynamic Variables and PI in Fluid Responders vs. Non-responders

Before VE, MAP, CO, SV, and HR did not differ between fluid responders and non-responders (Table 2). After VE, only SV increased in fluid responders while MAP and HR were not different before and after VE in fluid responders (Table 2). PI did not change after VE in both groups (Table 2). Changes in PI and SV in fluid responders and non-responders during VE are shown in Figure 1.

### Baseline Parameters at T1 and Changes in PI During LRM to Predict Fluid Responsiveness

**Figure 2:**
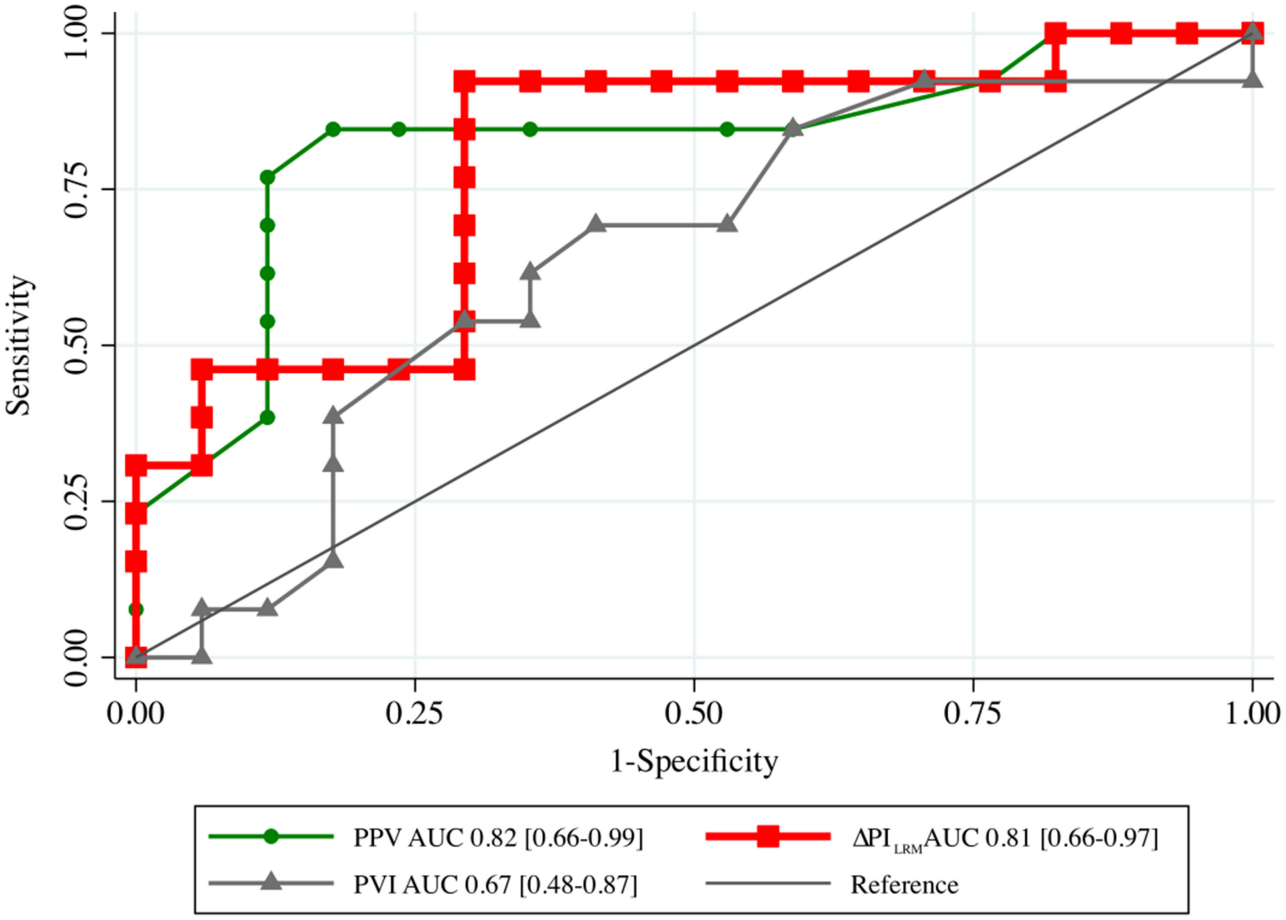
The area under the receiver operating characteristic (AUC) curves generated from the changes in PI following LRM (ΔPI_LRM_), PPV and PVI before lung recruitment maneuver (T1) to predict fluid responsiveness. LRM, lung recruitment maneuver; PPV, pulse pressure variation; PVI, pleth variation index.

ΔPI_LRM_ [0.81 (0.66–0.97)] and PPV_T1_ [0.82 (0.66–0.99)] showed higher AUCs than ΔCO_LRM_, ΔSBP_LRM_, ΔMAP_LRM_, PVI_T1_, and PI_T1_ to predict fluid responsiveness (Table 3; Figure 2) with cut-off values ≥40% [Sn of 92.3% (95% CI, 64.0–99.8%); Sp of 73.7% (95% CI, 48.8–90.9%); positive predictive value of 70.6% (95% CI, 44.0–89.7%); negative predictive value of 93.3% (95% CI, 68.1–99.8%); and LR+ of 3.51 (95% CI, 1.63–7.57)]. ΔPI_LRM_ had similar AUC with PPV_T1_, *p* = 0.806.

**Table 3:** The changes in hemodynamic parameters and their AUCs in predicting fluid responsiveness.

**Hemodynamic parameters**	**Fluid responders**	**Fluid non-responders**	**AUC**	**95% CI**
	**(*n* = 13)**	**(*n* = 19)**		
**A decrease in hemodynamic parameters following lung recruitment maneuver**				
ΔPI_LRM_ (%)	55.23 ± 17.82	35.32 ± 17.32	0.81	0.66–0.97
ΔSV_LRM_ (%)	49.42 ± 21.49	39.79 ± 21.44	0.65	0.45–0.86
ΔCO_LRM_ (%)	58.92 ± 18.70	47.39 ± 22.87	0.70	0.51–0.88
ΔSBP_LRM_ (%)	30.42 ± 11.56	20.22 ± 10.50	0.72	0.54–0.91
ΔDBP_LRM_ (%)	19.12 ± 10.59	14.55 ± 8.99	0.60	0.38–0.82
ΔMAP_LRM_ (%)	26.27 ± 10.93	19.47 ± 9.61	0.67	0.47–0.88
ΔHR_LRM_ (%)	5.08 ± 8.93	7.12 ± 7.50	0.43	0.22–0.64
**Respiratory variation of hemodynamic parameters at T1**				
PVI_T1_ (%)	13.92 ± 4.77	12.00 ± 6.82	0.67	0.48–0.87
PPV_T1_ (%)	18.85 ± 7.55	9.53 ± 6.00	0.82	0.66–0.99
SVV_T1_ (%)	25.91 ± 12.56	17.88 ± 6.28	0.69	0.46–0.92

*AUC, area under the curve; CI, confidence interval; CO, cardiac output; DBP, diastolic blood pressure; HR, heart rate; LRM, lung recruitment maneuver; MAP, mean arterial pressure; PI, perfusion index; PPV, pulse pressure variation; PVI, pleth variability index; SBP, systolic blood pressure; SV, stroke volume; SVV, stroke volume variation. ΔSV_LRM_; relative reduction rate of SV between T1 and T2, ΔCO_LRM_ relative reduction rate of CO between T1 and T2, ΔSBP_LRM_ relative reduction rate of SBP between T1 and T2, ΔDBP_LRM_ relative reduction rate of DBP between T1 and T2, ΔMAP_LRM_ relative reduction rate of MAP between T1 and T2, and ΔHR_LRM_ relative reduction rate of HR between T1 and T2*.

### Correlation of ΔPI_LRM_, PVI, PI, and PPV_T1_ With Changes in SV

**Figure 3:**
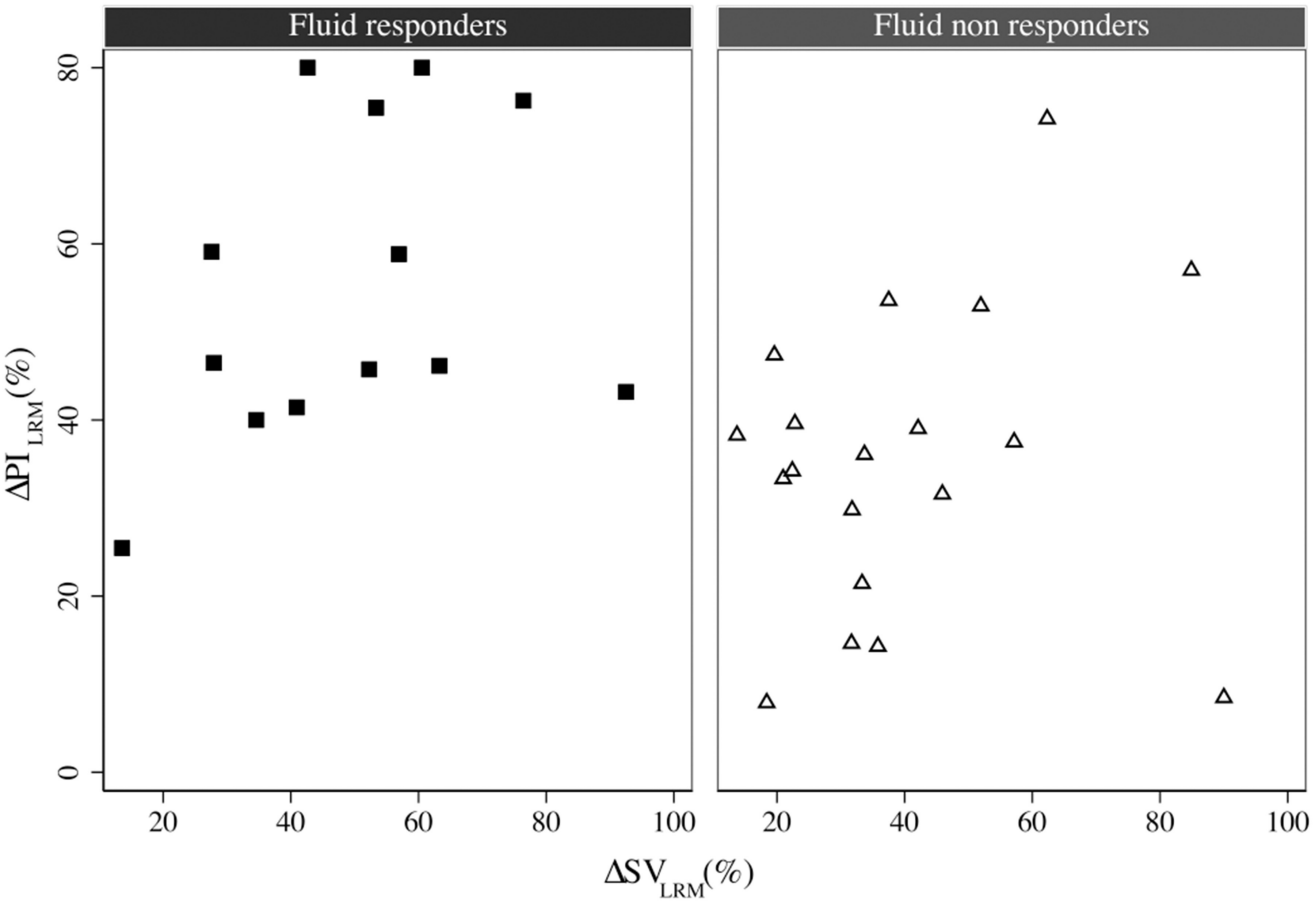
The correlation between ΔSV_LRM_ and ΔPI_LRM_ in fluid responders (square) and non-responders (triangle), *r*^2^ = 0.16, *p* = 0.020. LRM, lung recruitment maneuver; SV, stroke volume; PI, perfusion index.

ΔPI_LRM_ and PPV_T1_ showed significant correlations with ΔSV_VE_ (*r*^2^ = 0.36, *p* = 0.040 and *r*^2^ = 0.40, *p* = 0.028, respectively) and ΔSV_LRM_ (*r*^2^ = 0.16, *p* = 0.020 and *r*^2^ = 0.17, *p* = 0.038, respectively) (Figure 3). The relative change of PI and SV were correlated when considering all interventions (both LRM and VE) (*r*^2^ = 0.14, *p* = 0.028, concordance rate = 29.64%) (SDC, Figure 2). There was a significant correlation between PVI_T1_ and ΔSV_VE_ (*r*^2^ = 0.25, *p* = 0.036), but not with ΔSV_LRM_.

### The Gray Zone of ΔPI_LRM_

**Figure 4:**
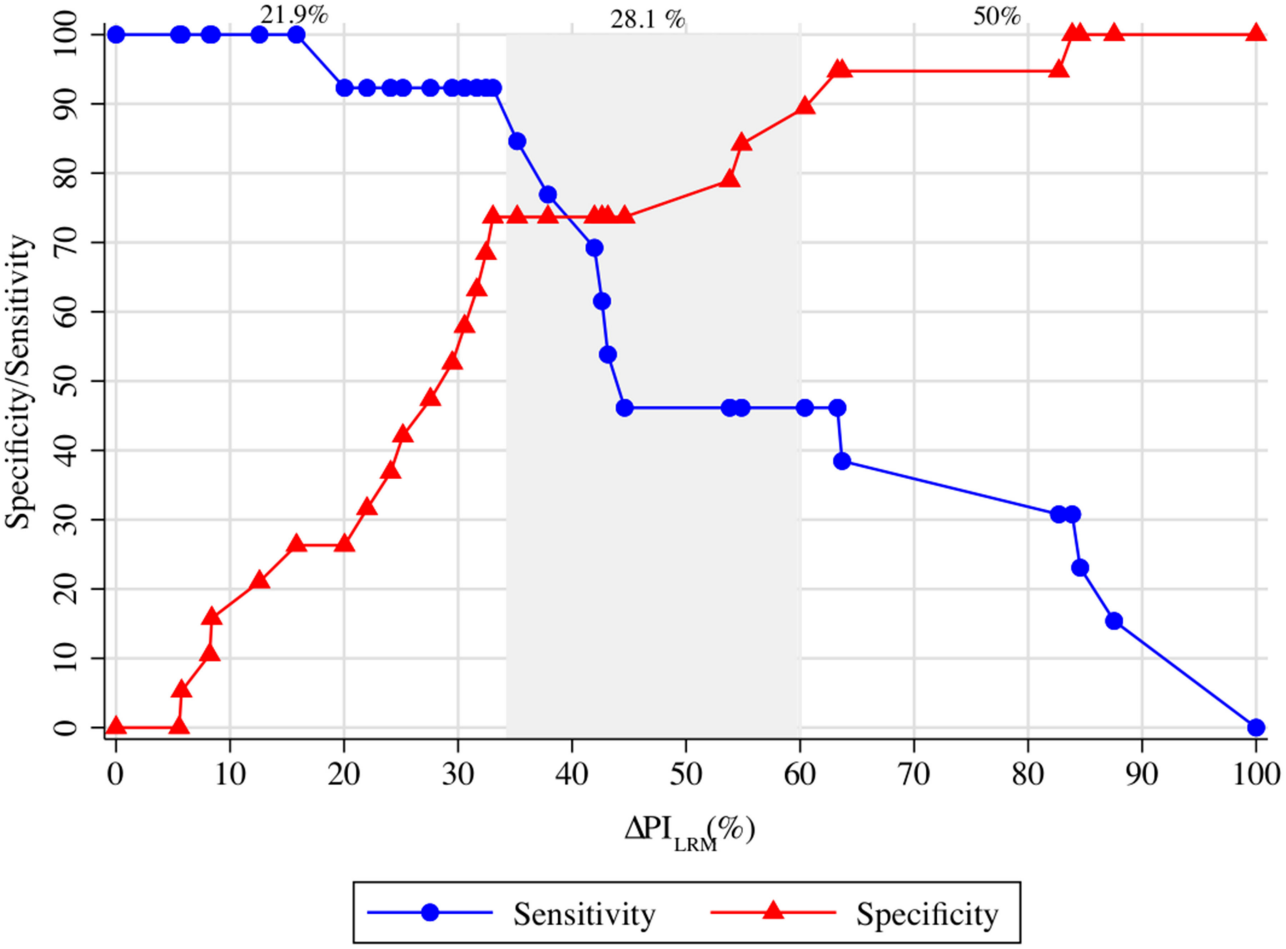
The gray zone approach of ΔPI_LRM_ and ΔSV_VE_. ΔPI_LRM_ <35.0% represents fluid non-responsiveness with a sensitivity >90%. Moreover, ΔPI_LRM_ >60.0% represents fluid responsiveness with a specificity >90%. The inconclusive zone of ΔPI_LRM_ is spread between 35.0 and 60.0%, which represents 28.1% of the population. LRM, lung recruitment maneuver; SV, stroke volume; PI, perfusion index.

Figure 4 depicts the gray zone approach for the ΔPI_LRM_. A decrease in ΔPI_LRM_ of <35% could guide the decision-making for fluid non-responders with a Sn ≥90%. In contrast, a decrease in ΔPI_LRM_ of more than 60% could detect fluid responsiveness with a Sp ≥90%. Over 21.9 and 50.0% of our population, respectively, could undergo guided decision-making by the ΔPI_LRM_ regarding whether to receive VE or not. However, in 28.1% of the population, this was inconclusive.

## Discussion

In this study, we demonstrated that a reduction in PI during LRM and the baseline PPV had better ability to predict fluid responsiveness in surgical patients who underwent elective open abdominal surgery than the baseline MAP, CO, and PVI.

Predicting fluid responsiveness plays an important role in optimizing perioperative fluid infusion. Inappropriate fluid administration including inadequate fluid infusion during perioperative period are related to the development of acute kidney injury or an increase in postoperative complications such as infection, which is associated with mortality (1–5). Dynamic hemodynamic parameters which rely on heart-lung interactions, such as PPV and SVV are better indicators to predict fluid responsiveness than static hemodynamic variables such as MAP or CVP (11–13). However, PPV and SVV are less reliable in patients under mechanical ventilator with tidal volume <6 mL/kg (15). To overcome these limitations, end-expiratory occlusion test (36) and LRM (17, 18) are applied to evaluate the dynamic response of PPV or SVV.

Biais et al. (18) and Watanabe et al. (17) showed that a decrease in SV after LRM indicate fluid responsiveness during perioperative period. However, a change in SV during LRM in this study did not differ between fluid responders and non-responders, and it did not indicate fluid responsiveness. This finding can be explained by the differences in the sites of operation and devices between their publications (17, 18) and our study. The patients in this present study underwent open abdominal surgery whereas those in Biais et al. (18) and Watanabe et al. (17) underwent neurological and spine surgery, respectively. Additionally, SV in their publications (17, 18) were obtained by pulse contour analysis while in this present study, it was derived by esophageal Doppler. LRM might interfere with the aortic signal, leading to SV (37).

The PI signal represents the peripheral perfusion and depends on the global blood flow (SV and CO) and peripheral vasomotor tone. Thus, low PI could indicate either vasoconstriction and/or low SV (26, 27). While, high PI suggests vasodilatory state or high CO (38). When performing a preload test, as vascular tone does not change during this transient test, changes in PI track changes in CO (28). Courson et al. (35) demonstrated that a reduction in PI after LRM can predict fluid responsiveness in patients undergoing neurological surgery. In this study, we showed that the ΔPI_LRM_ was related with changes in SV during LRM and after VE and ΔPI_LRM_ was a good indicator to predict fluid responsiveness in patients undergoing open abdominal surgery; similar to the baseline PPV. Therefore, these findings confirmed the use of PI to detect a change in SV during LRM or VE. Moreover, ΔPI_LRM_ can be applied in patients with mechanical ventilator who are not requiring arterial catheter to detect fluid responsiveness. Furthermore, in this study, ΔPI_LRM_ had better ability to indicate fluid responders than PVI at baseline. This finding is similar to that of a previous meta-analysis (39), which reported that PVI reliability to predict fluid responsiveness in surgical patients under mechanical ventilators might be reduced. Nevertheless, further studies should be performed to validate these findings. Regarding the reduction in MAP and SV, LRM should be performed with caution in patients with hypotension or those requiring vasopressors.

In this study, PI did not change after VE in both fluid responders and non-responders. Ryu et al. (40) reported that sevoflurane and desflurane affected the PI values by inducing vasodilatation. Patients in this study received sevoflurane or desflurane, suggesting that in these patients, PI might have affected various vasoplegia states because of the anesthetic agents. This might explain the unchanged PI values after VE in fluid responders and non-responders (40, 41).

In fluid non-responders, PI and SV also decreased; this may be explained by the negative effect of the increase in the intrathoracic pressure during LRM on hemodynamic variables (29, 30) and volume status (42), or the degree of vasoplegia due to the anesthetic agents (40, 41). Moreover, the decrease in SV in fluid non-responders was similar to that reported by Biais et al. (18) and Watanabe et al. (17).

Our study also had some limitations. The first related to the esophageal Doppler technique. The cross-sectional area of the descending aorta was not applied in our technique. Therefore, this could lead to an underestimation of SV (37). Although with some considerations, this technique remains acceptable for tracking the trending ability when comparing it with the pulmonary artery thermodilution technique (43). Second, we performed LRM on patients in the supine position who mostly underwent open-abdominal surgery. The study period with LRM were performed before the surgery started; therefore, the results cannot be inferred to patients in other positions or other clinical situations, including laparoscopic surgery. The utilization of such a high tidal volume (8.7 ± 0.7 mL/predicted kg) in our study may limit the applicability of our ΔPI_LRM_ in a low tidal volume setting (6–8 mL/predicted kg). Moreover, a higher tidal volume, rather than a lower tidal volume, could emphasize the effects of LRM and thus produce a greater ΔPI_LRM_, even in patients with good lung compliance and good transmission of pleural pressure. Therefore, confirming this hypothesis requires further investigations. Third, the sample size calculation did not take into account the accuracy of the esophageal Doppler to detect the changes in SV. Therefore, the number of participants in this study might be smaller than the actual required sample size. Fourth, an inconclusive zone of ΔPI_LRM_ between 35 and 60% needs further attention. Another test to predict fluid responsiveness is needed for these populations. Fifth, LRM in this study was performed shortly after an induction of the anesthetic agents; therefore, the effect of LRM on hemodynamic status might have been impacted by the degree of vasodilatation due to the anesthetic agents. Nevertheless, fluid administration in patients with various vasoplegia during anesthetic period should be based on clinical decision.

Despite these limitations, a reduction in PI after LRM obtained non-invasively by pulse oximetry can be applied as an indicator to predict fluid responsiveness in patients undergoing abdominal surgery, similar to the baseline PPV.

## Data Availability Statement

The original contributions presented in the study are included in the article/Supplementary Material, further inquiries can be directed to the corresponding author/s.

## Ethics Statement

The study protocol was approved by the Institutional Review Board of Human Research Ethics Committee of the Faculty of Medicine, Ramathibodi Hospital, Mahidol University, Bangkok, Thailand (COA. MURA2020/1844). The study was registered with the Thai Clinical Trials Registry, code TCTR20201202001 (http://www.thaiclinicaltrials.org/show/TCTR 20201202001), on 2 December 2020. The patients/participants provided their written informed consent to participate in this study.

## Author Contributions

SM and NP created the conception and designation of the study. PPer wrote and submitted the proposal. PPer and SM obtained the data. NP, SM, PPin, and KT analyzed and interpreted the results. PPer and PPin drafted the work. NP, SM, WM, and KT substantively revised the manuscript. SM provided the greatest contribution to the study. All authors read and approved the final version of the manuscript.

## Funding

Grant support for the study was provided by the Faculty of Medicine Ramathibodi Hospital, Mahidol University, Bangkok, Thailand. The funder had no role in the study design, data collection, analysis, interpretation of the data, and preparation of the manuscript. In addition, all equipments in this study were provided by the Department of Anesthesiology, Faculty of Medicine Ramathibodi Hospital.

## Conflict of Interest

The authors declare that the research was conducted in the absence of any commercial or financial relationships that could be construed as a potential conflict of interest.

## Publisher's Note

All claims expressed in this article are solely those of the authors and do not necessarily represent those of their affiliated organizations, or those of the publisher, the editors and the reviewers. Any product that may be evaluated in this article, or claim that may be made by its manufacturer, is not guaranteed or endorsed by the publisher.
