# Supplementary material for: Prediction of Fluid Responsiveness by the Effect of the Lung Recruitment Maneuver on the Perfusion Index in Mechanically Ventilated Patients During Surgery
Source: Front Med (Lausanne). 2022 Jun 17;9:881267. doi: 10.3389/fmed.2022.881267 (PMC9247540; doi:10.3389/fmed.2022.881267)
Supplement: Supplementary file 1 [file Table_1.DOCX]

Table S1 Clinical characteristics in fluid responders and fluid non-responders

| Variables | All patient (32) | Fluid responders (N=13) | Fluid non-responders (N=19) |
| --- | --- | --- | --- |
| Age (year) | 60.2 ± 9.5 | 61.0±8.6 | 59.7 ± 10.3 |
| Male, n (%) | 20 (62.5%) | 8 (62%) | 12 (63%) |
| Height (cm) | 160.0 ± 6.8 | 159.5±6.9 | 160.4±6.9 |
| Body weight (kg) | 60.4 ± 12.1 | 57.5±12.9 | 62.4± 11.4 |
| Predicted body weight (kg) | 55.3 ± 7.6 | 54.7±7.8 | 55.6±7.6 |
| Body mass index (kg/m^2^) | 23.5 ± 4.2 | 22.5±4.3 | 24.2±4.1 |
| Body surface area (m^2^) | 1.62 ± 0.16 | 1.59±0.16 | 1.64±0.16 |
| ASA physical status I/II/III, n | 2/15/15 | 0/5/8 | 2/10/7 |
| Operative sites, n (%) |  |  |  |
| Liver  Pancreases  Renal  Gynecology  Breast | 14 (43.8%)  11 (34.4%)  3 (9.4%)  3 (9.4%)  1 (3.0) | 7 (53.9%)  4 (30.8)  2 (15.4%)  0 (0%)  0 (0%) | 7 (36.8%)  3 (36.8%)  1 (5.26)  3 (15.8%)  1 (5.3%) |
| Tidal volume (ml) | 480 ± 48 | 471±47 | 486± 49 |
| Tidal volume/predicted body weight (ml/kg) | 8.7 ± 0.7 | 8.7± 0.7 | 8.8±.7 |
| Respiratory rate (breaths/min) | 12.8 ± 1.4 | 13.0±1.4 | 12.6±1.5 |
| Plateau pressure (cm H_2_O) | 16.1 ± 2.2 | 15.9±2.8 | 16.3± 1.9 |
| Driving pressure (cm H_2_O) | 11.2 ± 2.2 | 11.3±2.7 | 11.2±1.9 |
| Positive end expiratory pressure (cm H_2_O) | 5.0 ± 1.0 | 4.7±1.4 | 5.2± .05 |
| Vasopressor usage, n (%) | 5 (15.6%) | 3 (23.1%) | 2 (10.5%) |

Data was expressed as mean±SD, *p<0.05 between fluid responders vs fluid non-responders
