# Supplementary material for: Prediction of Fluid Responsiveness by the Effect of the Lung Recruitment Maneuver on the Perfusion Index in Mechanically Ventilated Patients During Surgery
Source: Front Med (Lausanne). 2022 Jun 17;9:881267. doi: 10.3389/fmed.2022.881267 (PMC9247540; doi:10.3389/fmed.2022.881267)
Supplement: Supplementary file 2 [file Image_1.pdf]

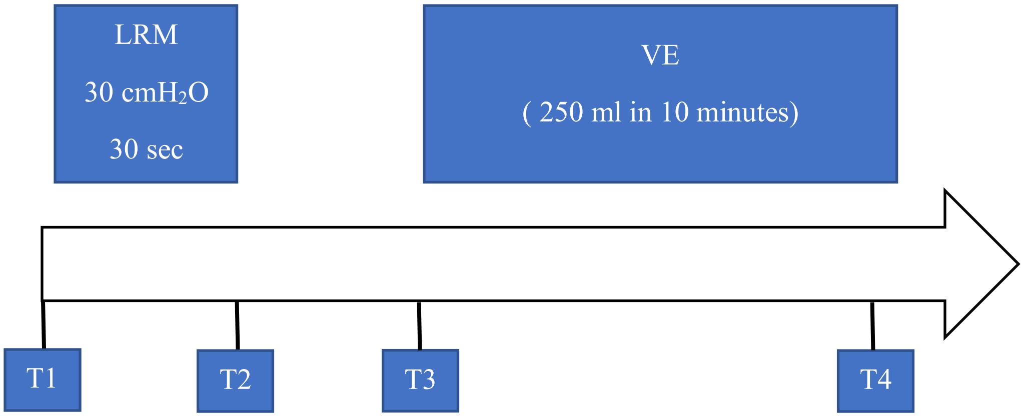

Figure 1S. Flow diagram to perform LRM and VE. Sets of measurements were performed before LRM (T1), after LRM (T2), immediately after SV and PI returned to their baseline values (variations less than 10%) (T3) and immediately after VE (T4).

Abbreviations LRM, lung recruitment maneuver; VE, volume expansion; SV, stroke volume; PI, perfusion index
