# Supplementary figures and images for: Prediction of Fluid Responsiveness by the Effect of the Lung Recruitment Maneuver on the Perfusion Index in Mechanically Ventilated Patients During Surgery
Source: Front Med (Lausanne). 2022 Jun 17;9:881267. doi: 10.3389/fmed.2022.881267 (PMC9247540; doi:10.3389/fmed.2022.881267)

Changes in Perfusion Index (%)

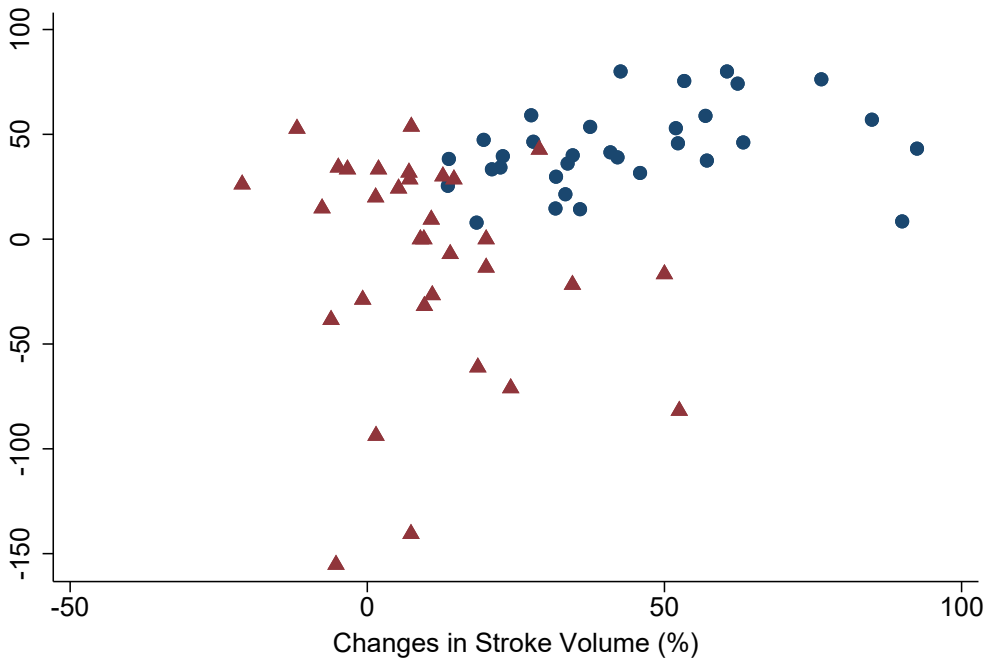

● Lung recruitment maneuver    ▲ Volume expansion

Supplement: Supplementary file 3 [file Image_2.pdf]
